# Demineralized human dentin matrix for alveolar ridge preservation using a volumetric and histologic analyses in rats

**DOI:** 10.1590/0103-6440202204648

**Published:** 2022-06-24

**Authors:** Gabriela Fracasso Moraes, Rodrigo de Oliveira Caetano, Fernanda Harumi Oku Prochnow, Yasmine Mendes Pupo, Juliana Lucena Schussel, Humberto Osvaldo Schwartz-Filho

**Affiliations:** 1 Post graduate Program in Dentistry, Department of Stomatology, Universidade Federal do Paraná, UFPR, Curitiba, Brazil; 2 Post graduate Program in Dentistry, Department of Restorative Dentistry, Universidade Federal do Paraná, UFPR, Curitiba, Brazil

**Keywords:** Demineralized human dentin matrix, biomaterial, biocompatibility, alveolar ridge preservation, bone substitutes

## Abstract

The aim of this study was to evaluate a Demineralized Human Dentine Matrix (DHDM) as viable biomaterial for alveolar ridge preservation in a rat model. Wistar rats were submitted to the extraction of maxillary first molars bilaterally. Sockets were filled with biomaterials and divided into 4 experimental groups (n=5): blood clot, autogenous bone, bovine-derived xenograft (BDX) and DHDM. Animals were sacrificed at 7, 14 e 28 days. Microtomography (uCT) volumetric evaluation and qualitative histological analyses were performed. Results obtained through the uCT showed similar values between the DHDM and the other experimental groups. The histological evaluation demonstrated DHDM with an unspecific inflammatory process and bone neoformation with slow reabsorption of the material. This result indicates that DHDM implanted in rat sockets is biocompatible and reduces the alveolar ridge volume loss after tooth extraction.

## Resumo

O objetivo deste estudo foi avaliar a Matriz Dentinária Humana Desmineralizada (MDHD) como biomaterial viável para preservação do rebordo alveolar, no modelo em rato. Ratos Wistar foram submetidos à exodontias dos primeiros molares superiores bilateralmente. Os alvéolos foram preenchidos com biomateriais e divididos em 4 grupos experimentais (n=5): coágulo sanguíneo, osso autógeno, osso xenógeno de origem bovina e MDHD. Os animais foram sacrificados aos 7, 14 e 28 dias. Foram realizadas avaliações volumétricas por microtomografia (uCT) e análises histológicas qualitativas. Os resultados obtidos por meio do uCT mostraram valores semelhantes entre o MDHD e os demais grupos experimentais. A avaliação histológica demonstrou MDHD com processo inflamatório inespecífico e neoformação óssea com lenta reabsorção do material. Esse resultado indica que a MDHD implantada em alvéolo de rato é biocompatível e reduz a perda de volume do rebordo alveolar após extração dentária.

## Introduction

Regardless of the reason why a tooth is extracted, remodeling of the alveolar process occurs as part of the repair process and is more pronounced in the vestibular wall of the maxillary alveolar processes, generating technical and aesthetic difficulties for oral rehabilitation ^1^ ^,^ ^2^. Several techniques and biomaterials have been evaluated to try to minimize this effect ^3^ ^,^ ^4^.

Alveolar socket/ridge preservation is the clinical procedure performed in an attempt to prevent resorption of post-extraction alveolar bone. Is an effective approach to attenuate/prevent horizontal bone resorption and buccal bone changes, maintaining the alveolar ridged form ^3^ ^,^ ^5^ ^,^ ^6^. Bone grafts and substitutes can be used alone or combined with some barrier, like membranes or screens. Ideally, biomaterials should be biodegradable, osteoconductive and provide mechanical resistance until the newly formed bone can maintain function ^7^.

Although the autogenous bone is considered the gold standard for bone reconstructions or for the preservation of the socket, it has disadvantages, such as volume limitation, greater morbidity, discomfort, pain and patient refusal ^8^. Thus, it is necessary to constantly search for new biomaterials that can replace autogenous bone grafts when necessary.

Bovine-derived xenograft (BDX) and Demineralized Human Dentin Matrix (DHDM) are alternatives to autogenous bone. Xenogenous and allogeneic grafts were introduced as a way to avoid greater aggression at the moment of the autogenous graft collection. Both bone and dentin are matrices of collagen fibers mineralized by hydroxyapatite. Although these tissues perform different functions, both have mesenchymal origin and are managed by mesenchymal cells that synthesize collagen and non-collagen proteins in different proportions. Studies show that these proteins can signal the induction of stem cell differentiation ^9^. In 2008, Dentin Morphogenetic Protein (DMP-1) was identified both in dentin and bone tissue ^10^. This relationship between the proteins presents in both dentin and bone led to research considering the possibility of the dentinal matrix as a bone substitute ^11^.

The clinical use of DHDM as a bone substitute was previously reported, including clinical trials ^5^ ^,^ ^12^. In extraction sockets exhibited minimal tissue response, bone regenerative potential and showed clinical efficacy comparable to that of BDX material, seeming to be a viable option. Even though insufficient data to support the histologic effects of DHDM in the alveolar bone repair process, studies in animals showed that DHDM induced a satisfactory and faster bone repair process ^13^ and presented proteins carrier potential ^14^.

Daily teeth are extracted and discarded, which can be collected and stored in a human tooth bank, generating an abundant and cheap source of dentin-based biomaterial to be used as an alternative to bone graft material ^15^. Different methods are described and can be used as a basis for obtaining the DHDM. The search for a preparation protocol and proof of its safety and reproducibility justify research in this field. The aim of this study was to evaluate, in a rat model, the potential of the DHDM as a bone substitute, preventing alveolar ridge volume loss after tooth extraction.

## Materials and methods

Demineralized human dentine matrix

Third molars were harvested from routine patients requiring their removal for clinical reasons at the Federal University of Parana. Soft tissue and periodontal ligament were completely removed with a curette ^16^. All the teeth were sent to the Biobank of human teeth at Universidade Federal do Paraná (BDH - UFPR) for processing. By grinding along the tooth profile, outer cementum and part of the dentin was removed. Dental pulp tissues and pre-dentin were removed. For size reduction of the granules, a grinder mill was used to obtain a granulation with of 500μm size ^7^. Chemical separation of the enamel, dentin and cementum structures was continued through centrifugation. Thus, the resulting human dentin matrix was moistened with deionized water for 5 h and mechanically cleaned for 20 min every hour using an ultrasonic cleaner. Enamel sedimentation and dentin remain in the supernatant phase. DHDM was obtained follows the sequence described for Li *et al* ^17^: 1) immersion in 17% Ethylene Diamine Tetra-acetic Acid (EDTA, Sigma, USA) solution for 5 min; 2) washing with deionized water for 10 min in an ultrasonic cleaner; 3) exposed to 10% EDTA for 5 min; 4) washing with deionized water for 10 min in an ultrasonic cleaner; 5) exposed to 5% EDTA demineralizing solution for 10 min, and final washing with deionized water water for 10 min in an ultrasonic cleaner and drying. As soon as this procedure was completed, the DHDM was sterilized by gamma irradiation. Gamma irradiation was performed in a gamma radiation chamber (Gammacell 220 Excel, GC-220E; MDS Nordion, Ottawa, Canada) for 18 h and 58 min at 27°C with a 14.5 kGy dose ^16^. Sterilized material was kept in cryopreservation at -80^o^C.

### Scanning electron microscopy (SEM) analysis

The particulate samples obtained in the study were seeded onto 6-well plates containing a microscope coverslip 13 mm in diameter and 0.13 mm thick (MODEL: G-13C/100, Glasscyto, Bioslide Technology, Walnut, Walnut, USA). The samples used were dried in an oven at 45ºC and deposited on stainless steel pieces located on the coverslip. Furthermore, were metallized with gold-palladium alloy (Au-Pd) using the DENTON VACUUM DESK V metallizer (Denton Vacuum, Moorestown, New Jersey, USA) with the aid of ADIXEN PASCAL 2005 SD (Ideal Vacuum Products, Albuquerque, New Mexico, USA), responsible for creating vacuum in the equipment. Afterwards, they were taken to the scanning electron microscope (SEM) sample chamber chamber (MODEL: 6010LA, Jeol®, Akishima, Tokyo, Japan) for surface analysis and characterization. Digital images were obtained at 500, 1000 and 5000x magnification and acceleration voltage of 10kV.

### Animals

This study was carried out with the approval of the Ethics Committee on the Use of Animals of the Federal University of Paraná (CEUA, Process 23075.163431/ 2016-83, Approval No. 1046). Wistar rats weighing approximately 150g were used. Animals were kept in rooms with 12 h light / dark cycle and controlled temperature (22-25^o^C), in cages with food and water ad libitum.

### Surgical procedures

Animals were submitted to general anesthesia by intraperitoneal injection with Ketamine Hydrochloride solution (Cetamin 10%, Syntec, 50 ml) and Xylazine Hydrochloride (Xilazin 2%, Syntec, 10 ml) in the proportion of 0.7 ml/g of Ketamine and 0.5 ml/g of Xylazine. First upper molars were extracted bilaterally, and the alveoli were separated randomly in 4 groups (n=5) to receive: blood clot; autogenous bone, BDX, and DHDM. It was gently accommodated with an instrument compatible with the size of the socket. Autogenous bone was harvested from calvaria of the same animals using a trephine bur. All animals were submitted to the same procedures. BDX commercial product, purchased from an authorized dealer (Bio-Oss® L: 1mm-2mm [0,5 g~1,5 cc; 2 g~ 6 cc], Geistlich Pharma do Brasil).

Analgesic medication (Paracetamol - Johnson & Johnson® do Brasil) at a dose of 15mg/kg in a single postoperative dose and were administrated. Euthanasia was performed after 7, 14 and 28 days using an overdose with an association of ketamine hydrochloride and xylazine hydrochloride. Maxilla were dissected and initially stored formaldehyde solution for fixation. After seven days, the pieces were removed from the formaldehyde solution and immersed in 98^o^ alcohol where they were kept until µCT analyses and histological processing.

### Microcomputed tomography (uCT)

The specimens were submitted to tomographic examination in a uCT device, model SkyScan 1172 (Bruker, Belgium) from the Laboratory of Mineral Analysis, Lamir, from the Federal University of Paraná. Examination was carried out according to the following standardized regimen: isotropic voxel of 12.89 μm, 90 kV, 112 μA at 180O, rotation angle of 0.6 and filter 0.5 mm with an exposure time of 1100 milliseconds. The images were reconstructed with specific NRECON® software (Bruker, Belgium) in about 1336 rows X 2000 columns. For three-dimensional evaluation and quantitative analysis, the CTAN® software, provided by the SkyScan® system, was used. The parameters analysed in this study were: bone volume (BV), percentage of bone volume (BV/TV) and bone surface (BS).

### Qualitative histological analyses

After uCT analysis, the same specimens were submitted to conventional histological processing and stained with Hematoxylin and Eosin (HE). After fixation in formaldehyde the specimens were decalcified in formic acid solution and formalin (40:60 proportion) for 15 days. After descaling, each specimen was sectioned in two blocks in the vestibulo-palatal direction, along the center of the socket (original surgical defect). The fragments were embedded in paraffin and serial cross sections of 5 μm thick at every 10 μm were prepared. The slides were then stained with HE. Qualitative analysis of the samples was carried out through evaluation of tissue repair and inflammatory infiltrate as well as bone formation in each group in the different times of the study. This analysis also aimed to assess the prevalence of inflammatory cells types as an indicator of repair and biocompatibility. A single experienced examiner that was blind for groups and times of the specimens analyzed all images.

### Statistical analysis

Statistical analyses were carried with GraphPad Prism program (version Prism 8 for Mac OS X, San Diego) using the non-parametric Kruskal-Wallis test for independent samples. If the result of the Kruskal-Wallis test was ‘significant’, i.e. occurrence of at least one significant difference (P<0.05). Dunn's multiple comparison post-test was performed.

## Results

### Scanning electron microscopy analysis

**Figure 1:**
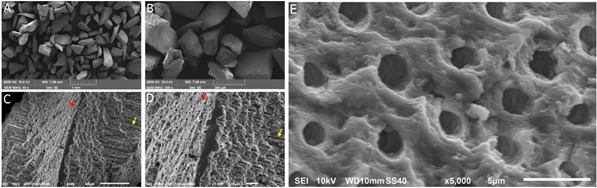
Scanning Electron Microscopy images of the Demineralized Human Dentine Matrix (DHDM). A) panoramic view of the DHDM particles (50x magnification) showing size homogeneity; B) approximate view of the particles (300x magnification), showing their irregular shape; C) photomicrograph of a DHDM particle (500x magnification). Red arrows show the transversely exposed dentinal tubules exposed transversely and yellow arrows show dentinal tubules exposed longitudinally; D) photomicrograph of a DHDM particle (1000x magnification). Red arrows show the transversely exposed dentinal tubules and yellow arrows show dentinal tubules exposed longitudinally and E) photomicrograph of the DHDM surface at magnification of 5000x.

The images obtained from the SEM are shown in figure 1. The DHDM particles obtained presented irregular topography, a result of the grinding process. It is possible to observe, also, the homogeneity in size distribution of the particles, demonstrating the effectiveness in the separation through the graduated screen sieves employed in this study (Fig 01 A and B). Dentinal tubules exposed both transversally and longitudinally as a result of the irregular shape of the particles (Fig 01 C and D). The EDTA was used to remove the smear layer and promote the superficial demineralization of dentin, resulting in the angular format of the DHDM particles. The demineralization protocol employed results in an irregular surface topography, as can be evidenced by figure 1 E. The erosive action of EDTA allowed the exposure of the intertubular and peritubular dentin, as well as the opening of the dentinal tubules. It can also be observed undulations within the tubules and on the dentin surface, suggestive of collagen fiber exposure.

### Computed microtomography

###  Bone volume 

**Figure 2:**
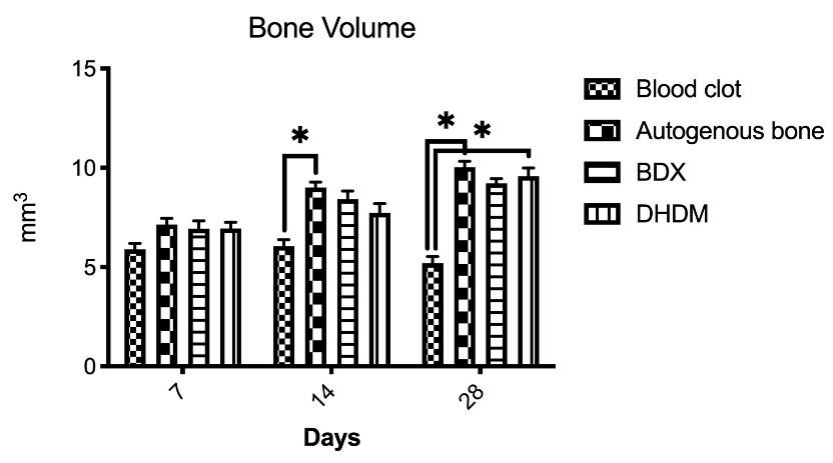
Bone volume (BV) (mm^3^) for all groups (blood clot, autogenous bone, bovine-derived xenograft [BDX] and Demineralized Human Dentine Matrix [DHDM]) evaluated at 07, 14 and 28 days. The bars represent the mean ± standard deviation of BV. p> 0.05 and 95% significance level (Kruskal-Wallis, Dunn's multiple comparison post-test, p <0.05). * Represent statistically significant differences between groups.

The results obtained through volumetric analysis by uCT are shown in the figure 2. The intergroup evaluation for 7 days period showed no differences (Kruskal-Wallis, p > 0.05). For the 14 days, there was a statistically difference between blood clot and autogenous (Kruskal-Wallis, Dunn's multiple comparison post-test, p = 0.009). At 28 days, there was significant difference, BV was higher in autogenous and DHDM group when compared with blood clot (Kruskal-Wallis, Dunn's multiple comparison post-test, p = 0.01).

###  Percentage of bone volume 

**Figure 3:**
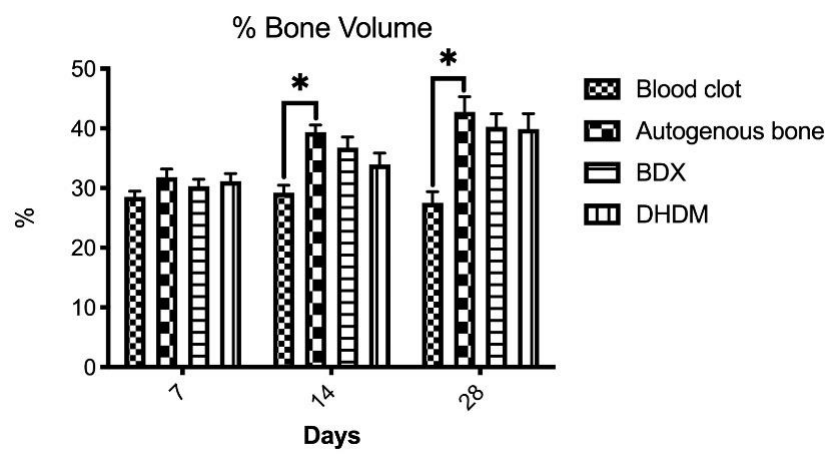
Percentage of bone volume (BV/TV) (%) for all groups (blood clot, autogenous bone, bovine-derived xenograft [BDX] and Demineralized Human Dentine Matrix [DHDM]) evaluated at 07, 14 and 28 days. The bars represent the mean ± standard deviation of BV/TV. p> 0.05 and 95% significance level (Kruskal-Wallis, Dunn's multiple comparison post-test, p <0.05). * Represent statistically significant differences between groups.

The results obtained through volumetric analysis by uCT are shown in the figure 3. The intergroup evaluation for 7 days period showed no differences (Kruskal-Wallis, p > 0.05). Statistically significant differences were observed between blood clot and autogenous group for 14 as for 28 days periods. Bone density in the autogenous group was higher compared to blood clot (Kruskal-Wallis, Dunn's multiple comparison post-test, p = 0.01). The DHDM and BDX groups did not present statistically significant results for the other evaluated groups (Kruskal-Wallis, p > 0.05).

###  Bone surface 

**Figure 4:**
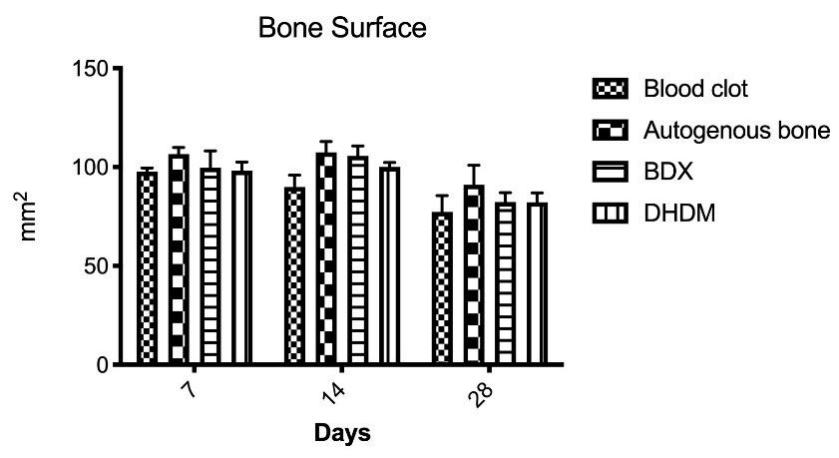
Bone surface (BS) (mm^2^) (%) for all groups (blood clot, autogenous bone, bovine-derived xenograft [BDX] and Demineralized Human Dentine Matrix [DHDM]) evaluated at 07, 14 and 28 days. The bars represent the mean ± standard deviation of BS. p> 0.05 and 95% significance level (Kruskal-Wallis, Dunn's multiple comparison post-test, p <0.05). No statistically significant results for the evaluated groups.

The results obtained through volumetric analysis by uCT are shown in the figure 4. For this parameter, no difference was observed in any of the evaluated periods (Kruskal-Wallis, p > 0.05).

### Qualitative histological analyses

For all groups and times analyzed, there were no signs of an infectious process or bone necrosis.

###  7-day period 

In general, all groups presented in 7 days tissue compatible with repair (Figure 05). Dense connective tissue with intense inflammatory lymphocytic infiltrate. In the region, it was possible to verify cortical bone in the peripheral region and central portion with a small amount of trabecular bone, with osteoblastic activity and bone matrix. Autogenous, BDX, and DHDM showed a fragment of material partially covered by mucosa. It was possible to observe mineralized dental tissue in the DHDM group (Figure 06).

###  14-day period 

It was possible to observe viable bone in the maturation process, with a predominance of trabecular bone with osteoblastic activity and a highly cellularized bone matrix (Figure 07). For the BDX group fragments of mineralized material compatible with biomaterial were also observed in two samples. In four samples, fragments of dentinal tissue were observed for DHDM.

**Figure 5:**
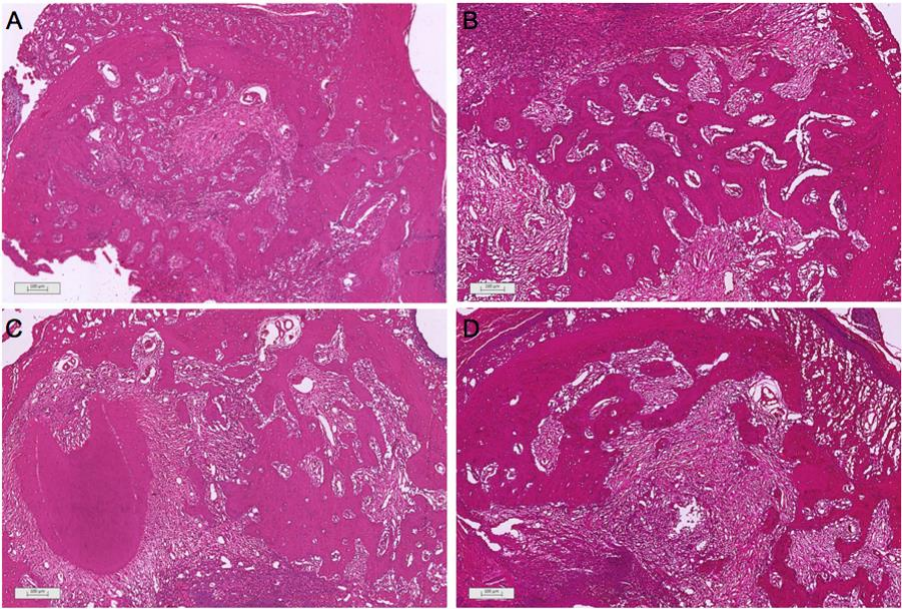
Histological sections of socket repair after extraction for the period of 7 days. A) blood clot; B) autogenous bone; C) bovine-derived xenograft (BDX) and D) Demineralized Human Dentine Matrix (DHDM).

**Figure 6:**
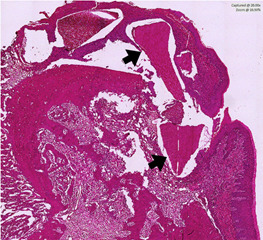
Histological section of the Demineralized Human Dentine Matrix (DHDM) for the period of 7 days. It is possible to observe mineralized dental tissue in the DHDM (Arrow).

**Figure 7:**
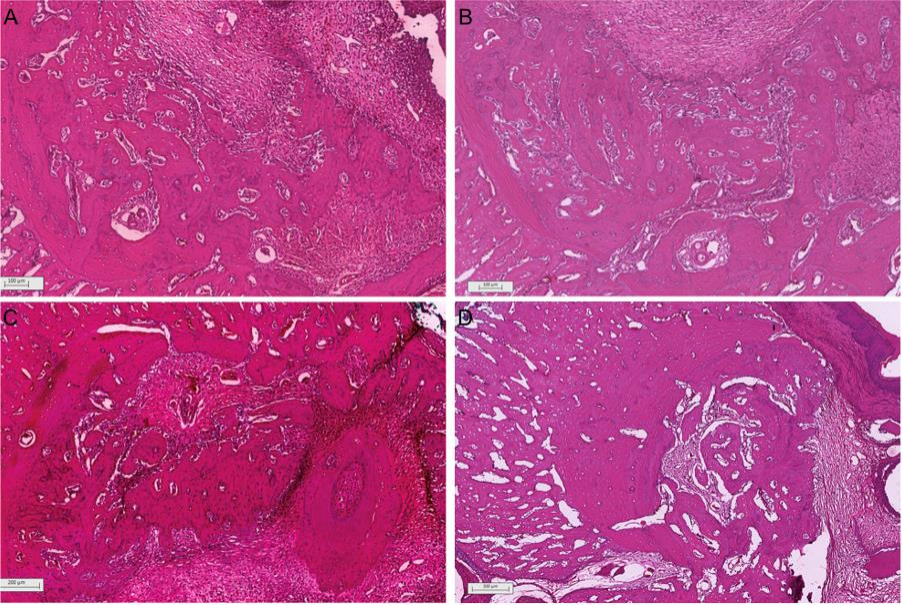
Histological sections of socket repair after extraction for the period of 14 days. A) blood clot; B) autogenous bone; C) bovine-derived xenograft (BDX) and D) Demineralized Human Dentine Matrix (DHDM).

###  28-day period 

**Figure 8:**
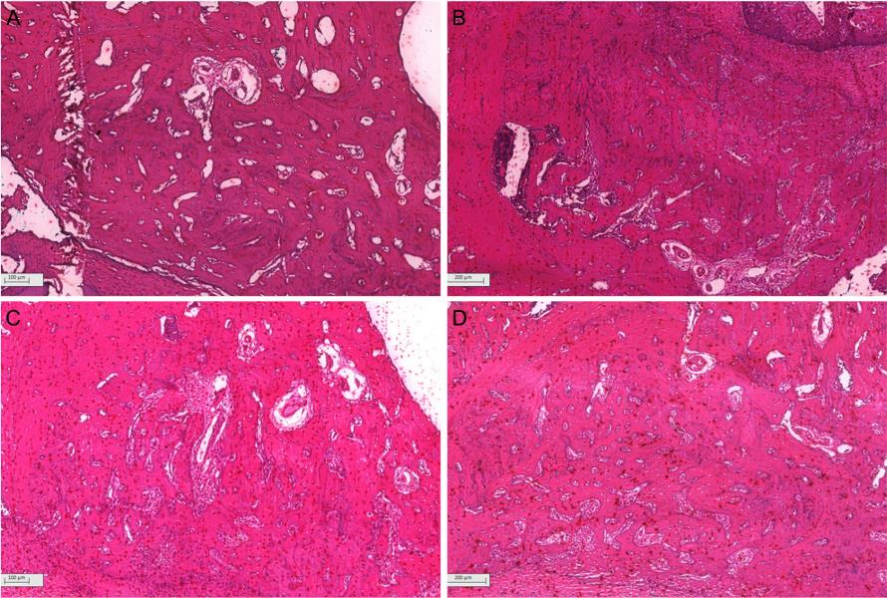
Histological sections of socket repair after extraction for the period of 28 days. A) blood clot, B) autogenous bone, C) bovine-derived xenograft (BDX) and D) Demineralized Human Dentine Matrix (DHDM).

**Figure 9:**
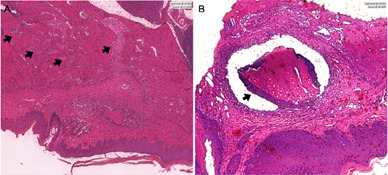
Histological sections of socket repair after extraction for the period of 28 days. A) Moderate inflammatory infiltrate in bovine-derived xenograft (BDX) within 28 days (Arrow); B) Dentin remnants in Demineralized Human Dentine Matrix (DHDM) (Arrow).

In general, all groups presented alveolar bone partially covered by hyperparakeratinized squamous mucosa. Lamina propria constituted by dense connective tissue showed a discrete lymphocytic inflammatory infiltrate compatible with the phase of inflammatory process and repair. It was possible to observe viable bone in the maturation phase, with a predominance of trabecular bone with osteoblastic activity and a highly cellularized bone matrix (Figure 8). Moderate inflammatory infiltrate in BDX group and dentin remnants in DHDM group were observed within 28 days (Figure 9).

## Discussion

The use of the dentin matrix as a biomaterial for bone formation has been proposed since the late 1960s with studies of the homogeneous dentin matrix and induction of ectopic bone formation in muscle, skin, bone marrow, cartilage and periodontal ligament without immune rejection from host ^18^. The process of preparing the experimental DHDM by BDH - UFPR is based on the work of Li, et al ^17^ who evaluated the potential for dentin regeneration through a fully demineralized dentin matrix in an animal model.

There is no consensus on dentin demineralization. While some authors find better results with partially demineralized dentin ^19^, others indicate total matrix demineralization ^20^. In addition to the degree of demineralization, another factor to be considered when preparing the dentinal matrix is the size of the final particle. While the study by Koga, et al ^19^ suggests the use of large particles in the size of 1000 μm, the study by Nam, et al ^21^ indicates particles of size between 250μm to 1000 μm, but emphasizes the need to create a space between the particles so that the framework formed by the matrix has vascular permeability. Thus, in this study it was decided to use particles of intermediate size of 500μm.

However, allogeneic and xenogenous grafts may present several limitations, including rejection, risk of strong antigenic reaction, transmission of infectious diseases, and requirement of adequacy between donor and recipient of bone tissue. For their use to be safe with respect to antigenicity, they must undergo previous processing by freezing, radiation or chemical ^22^. Demineralization has been confirmed as a viral inactivation method and is used by many tissue banks to validate viral clearance ^5^ ^,^ ^23^.

Results obtained from histology in the 7-day phase show, for all groups, same response described in literature with the presence of clot, inflammatory cells, beginning of angiogenesis process, migration and proliferation of mesenchymal cells, presence of fibroblasts and osteoblasts, an alveolus covered by epithelium and bone formation in the apical region. For the intermediate period of 14 days, the angiogenesis process was expected to become more evident, with the presence of migration and proliferation of mesenchymal cells, reduction of the inflammatory infiltrate, large number of fibroblasts and also small presence of active bone cells ^24^. However, for blood clot group an intense inflammatory infiltrate was still observed. For BDX group in this intermediate period, as expected, fragments of the grafted biomaterial were verified ^25^. It represents slow reabsorption, maintaining its osteoconductive potential, however, delaying the process of bone neoformation. Likewise, in the DHDM group, signs of dental material were verified, suggesting that this material also presents a slow resorption process. In the final period of 28 days of histological evaluation, all groups presented, as expected, viable bone formation with evidence of osteoblastic cells activity.

Several biomaterials have been evaluated for their ability to maintain the vestibular volume of the socket. Among them are: autogenous bone, different hydroxyapatites, xenogen bone, including dentin-based biomaterials ^3^ ^,^ ^4^ ^,^ ^5^ ^,^ ^6^. A particularity in our study should be highlighted, this tooth-derived graft cannot be considered as of autogenous origin or allogenic. Since it was obtained from humans and evaluated in rats, in this specific case, it should be considered as a xenogeneic material.

The findings of this study corroborate with that spontaneous repair, that is, just maintaining the clot in the alveolus, is not enough to maintain the volume bone tissue after tooth removal. However, authors argue that post-extraction reabsorption with the preservation procedure alveolar can be limited, but not entirely eliminated ^1^ ^,^ ^2^ ^,^ ^6^. Inspired by biomaterials produced from the dentin for bone grafting for alveolar preservation procedures, was demonstrated osteoinductive properties of DHDM ^13^. Furthermore, although that many biomaterials are capable of maintaining the morphology of the alveolar ridge, histologically, this result may not be translated into new bone formation.

Despite the fact that autogenous bone is considered the gold standard for intraoral grafts, including for the alveolar preservation procedure, there is a need to search for alternative materials, either due to the little availability, greater morbidity, pain and discomfort of the patient or difficulty to perform the technique ^5^ ^,^ ^8^. DHDM results were statistically similar to the autogenous bone and BDX in all evaluated uCT parameters (BV, BV/TS, BS). In the histological results, it demonstrated bone formation without adverse reactions.

Our results suggest that Demineralized Human Dentin Matrix produced by BDH - UFPR can be considered a biocompatible material, and was able to minimize post-extraction alveolar ridge volume loss in a rat model.
